# The influence of glyoxalase 1 gene polymorphism on its expression at different stages of breast cancer in Egyptian women

**DOI:** 10.18632/genesandcancer.163

**Published:** 2017-11

**Authors:** Rehab S. Abdul-Maksoud, Walid SH. Elsayed, Rasha S. Elsayed

**Affiliations:** ^1^ Medical Biochemistry Department, Faculty of Medicine, Zagazig University, Egypt; ^2^ Pathology Department, Faculty of Medicine, Zagazig University, Egypt; ^3^ General Surgery Department, Faculty of Medicine, Zagazig University, Egypt

**Keywords:** breast cancer, gene polymorphism, gene expression, enzymatic activity

## Abstract

**Aim:**

To assess the association of GLO1 C332C gene polymorphism with breast cancer risk at different stages of the disease and to investigate the effect of this gene polymorphism on its mRNA expression and enzyme activity.

**Methods:**

GLO1 C332C gene polymorphism was analyzed by PCR-RFLP in 100 healthy controls and 200 patients with breast cancer (100 patients with stage I & II and 100 patients with stage III & IV). GLO1 mRNA expression was measured by real time PCR. Serum GLO1 enzyme activity was measured colorimetrically.

**Results:**

GLO1 A allele was associated with increased risk of breast cancer [OR (95%CI)= 2.8(1.9-4.1), *P* < 0.001]. Its frequency was significantly higher among advanced stages of breast cancer compared with localized tumors (OR (95%CI)= 1.9(1.3-2.9), *p* < 0.001). GLO1 mRNA expression and enzyme activity were significantly higher in breast cancer patients compared to controls and they were much higher in the advanced stages of the disease (*P* < 0.001). Carriers of AA genotype showed higher GLO1 expression and enzyme activity compared with carriers of CC genotype.

**Conclusion:**

GLO1 C332C SNP was associated with overexpression of GLO1 mRNA and higher enzyme activity in breast cancer patients suggesting its role in the development of breast cancer and its progression from localized to advanced.

## INTRODUCTION

Breast cancer is the most common cause of cancer related mortality and represents the most frequent malignancy in women [1]. The high prevalence of breast cancer provides a strong rationale for identifying new molecular targets that can be modulated. Oxidative stress might have important roles in breast cancer development and progression [2]. Consequently, gene polymorphisms of antioxidant and antiglycation enzymes might affect individual susceptibility to breast cancer [3].

The antioxidant systems against oxidative and carbonyl stresses include glyoxalase I (GLO1) [4]. GLO1 is responsible for the detoxification of Methylglyoxal (MG), a byproduct of glycolysis. GLO1 catalyzes the binding of MG to reduced glutathione forming S-lactoyl- glutathione [5]. GLO1 gene is located on chromosome 6 (locus 6p21,3 - 6p21,2) [6]. A single nucleotide polymorphism at nucleotide position 332 (rs4746) of GLO1 causes adenosine/cytosine exchange in exon 4 of mRNA with subsequent amino acid change from Ala to Glu in position 111 in the protein sequence [7].

Methylglyoxal is responsible for the production of advanced glycation end products (AGEs) by modifying protein amino groups. AGEs can result in the production of free radicals and reactive oxygen species (ROS) [8]. Moreover, MG has the ability to attack DNA leading to DNA glycation [9]. Proliferation of cell lines and malignant transformation may occur if these changes involved proto-oncogenes or tumor suppressor genes, with subsequent mutagenesis and carcinogenesis [10].

Although the expression of GLO1 has been reported in some human cancers [11], very few studies were performed in selected population on GLO1 expression in breast cancer [12]. No data is available about this gene polymorphism and its expression in a rarely analysed ethnic cohort from Egypt. To the best of our knowledge we are the first to analyze the effect of GLO1 C332C gene polymorphism on its mRNA expression and enzyme activity at different stages of breast cancer in Egypt. Therefore, we hypothese that GLO1 C332C gene polymorphism with the resulting alteration in its expression might represent a promising target for the diagnosis of breast cancer.

## RESULTS

### Association of GLO1 gene with breast cancer risk

The genotype and allele frequencies of the GLO1 gene polymorphism in all studied groups were presented in Table 2. GLO1 SNP was in Hardy-Weinberg equilibrium both in patients and controls. SNP of GLO1 was associated with an increased risk of breast cancer. Increased risk of breast cancer was observed with the homozygous AA genotypes of GLO1 when compared with the CC genotype carriers [OR: 6.6(3.1-14), p < 0.001]. The frequency of GLO1 A allele was significantly higher in breast cancer patients compared to controls [OR: 2.8(1.9-4.1), p < 0.001].

When stratified by tumor stage, the frequency of the homozygous mutant genotype AA of GLO1, was significantly higher among advanced stages of breast cancer compared with less invasive tumors (p = 0.002). The frequency of the A allele of GLO1 was significantly higher among advanced cases of breast cancer compared with less invasive tumors (63 vs 46.5%, p = 0.001) (Table 2).

**Table 1:** Characteristics of breast tumors

Characteristics	No (%)
ER	
+ve	158(79)
-ve	42(21)
PR	
+ve	101(75)
-ve	99(25)
HER2	
+ve	38(19)
-ve	162(81)
Histology	
Ductal carcinoma in situ	15(7.5)
Invasive ductal carcinoma Lobular	175(87.5)
carcinoma in situ	1(0.5)
Invasive lobular carcinoma	9(4.5)
Subtypes	
Luminal A	70(35%)
Luminal B	98(49%)
HER2 overexpression	24(12%)
Triple negative	8(4%)
Tumor grade	
1	12(6%)
2	115(57.5)
3	80(36.5)
Clinical stage	
I	38(19)
II	62(31)
III	61(30.5)
IV	39(19.5)

**Table 2:** Association of GLO1 gene polymorphism with breast cancer risk

	Control (N=100) N(%)	Breast cancer (N=200) N(%)	P value*	OR (95%CI)	Localized BC (N=100) N(%)	P value*	OR(95%CI)	Advanced BC (N=100) N(%)	P value*	OR (95%CI)	P value**	OR (95%CI)
Genotype												
CC	51(51)	45(22.5)		1	31(31)			14(14)		1	0.05	1
CA	38(38)	91(45.5)	0.001	2.7(1.6- 4.7)	45(45)	0.05	1.9(1.1-3.6)	46(46)	<0.001	4.4(2.1- 9.2)	0.05	2.3(1.1- 4.8)
AA	11(11)	64(32)	<0.001	6.6(3.1- 14)	24(24)	0.004	3.6(1.5-8.3)	40(40)	<0.001	13.2(5.4- 2.3)	0.002	3.7(1.6- 2.4)
Allele												1
C	140(70)	181(45.25)		1	107(53.5)		1	74(37)		1		1
A	60(30)	219(54.75)	<0.001	2.8(1.9- 4.1)	93(46.5)	0.001	2(1.3-3.1)	126(63)	<0.001	3.9(2.6-6)	0.001	1.9(1.3- 2.9)

*P value when compared to control

P** when advanced breast cancer compared to localized group

The frequency of GLO1 genotypes and alleles didn't differ between different breast cancer subtypes as shown in Table 3.

**Table 3:** Association of GLO1 gene polymorphism with different breast cancer subtypes

	Luminal A (N=70) N(%)	Luminal B (N=98) N(%)	Her 2 (N=24) N(%)	Triple Negative (N=8) N(%)	P value
Genotype					
CC	15 (21.43)	24(24.48)	5(20.84)	1(12.5)	1.00
CA	36 (51.43)	41(41.84)	9(37.5)	5(62.5)	0.52
AA	19 (27.14)	33(33.67)	10(41.67)	2(25)	0.76
Allele					
C	66(47.14)	89(45.41)	19(39.58)	7(43.75)	1.00
A	74(52.86)	107(54.59)	29(60.42)	9(56.25)	

### GLO1 mRNA expression in breast cancer

**Figure 1:**
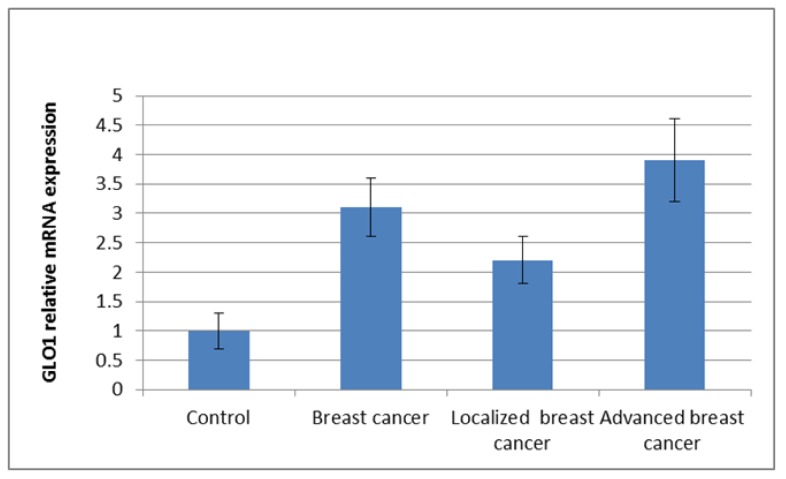
GLO1 mRNA expression by real time PCR among studied groups Data presented as mean±SD.

**Figure 2:**
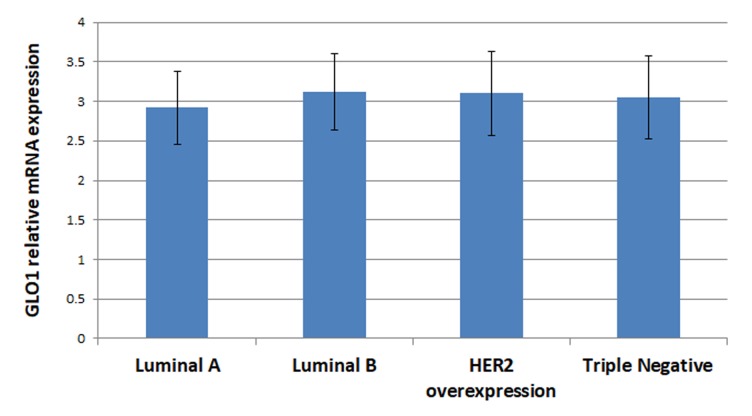
GLO1 mRNA expression by real time PCR among different subtypes of breast cancer Data presented as mean±SD.

GLO1 mRNA expression was presented as mean±SD, it was higher in breast cancer patients (3.1±0.5) compared to controls (1±0.3). The increase was much higher in advanced stages of breast cancer (3.9±0.7) (Figure 1). GLO1 overexpression does not correlate with different breast cancer subtypes, neither luminal A, Luminal B, Her2+ or TNBC (2.92±0.46, 3.12±0.48, 3.1±0.53 and 3.05±0.52 respectively; P = 0.06) (Figure 2).

**Figure 3:**
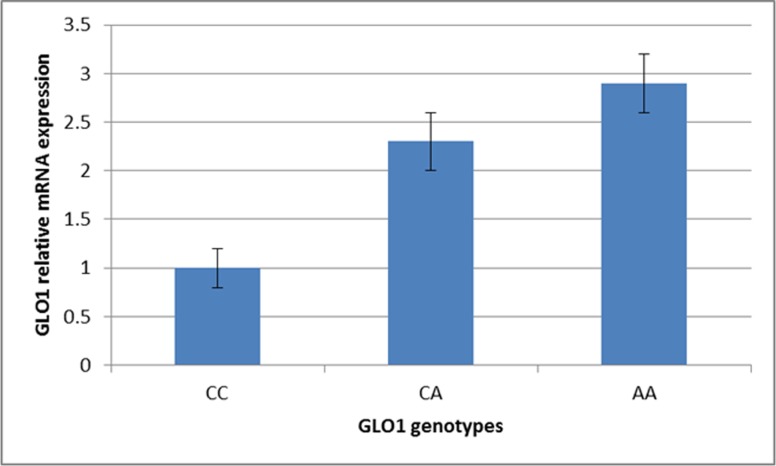
GLO1 mRNA expression by real time PCR among different GLO1 genotypes Data presented as mean±SD.

GLO1 mRNA was up-regulated with homozygous AA genotype (2.9±0.3) when compared with the CC genotype (1±0.2) or CA genotype carriers (2.3±0.3) (Figure 3).

### Association of GLO1 serum activity with breast cancer risk

Table 4 showed the mean values of GLO1 activities in the serum among all groups.

**Table 4:** Serum GLO1 enzyme activity in control and breast cancer patients

	Control (n=100)	Breast cancer (n=200)	P*	Localized breast cancer (N=100)	P*	Advanced breast cancer (N=100)	P*	P**
GLO1 umol/min	150±41	230±60	<0.001	200±50	<0.001	225±56	<0.001	0.001

P* when compared to control group

P** when advanced breast cancer compared to localized group

Regarding the serum activity of GLO1, breast cancer patients had higher serum GLO1 activity (230±60) compared to controls (150±41; p < 0.001). Patients with advanced breast cancer (225±56) had significantly higher GLO1 activity not only compared to controls, but also compared to localized breast cancer (200±50; P < 0.001) (Table 4).

There were non-statistically significant differences in GLO1 enzyme activity among different breast cancer subtypes (P = 0.6) (Table 5).

**Table 5:** Serum GLO1 enzyme activity in different breast cancer subtypes

	Luminal A (N=70)	Luminal B (N=98)	Her 2 (N=24)	Triple Negative (N=8)	P value
GLO1 umol/min	224±40	223±59	240±61	233±65	0.60

Among controls and different stages of breast cancer, individuals carrying AA genotype had a higher specific enzymatic activity compared to individuals with CC or CA genotype carriers (*P* < 0.001) (Table 6).

**Table 6:** Variation in serum enzyme activity by GLO1 C332C gene polymorphism

GLO1 activity umol/min	CC	CA	AA	P value
Control	101±31	163±32	199±57	<0.001
Localized breast cancer	154±30	209±43	232±74	<0.001
Advanced breast cancer	198±59	224±68	254±43	<0.001

P value calculated by ANOVA test

## DISCUSSION

Breast cancer represents the most frequent malignancy and the most common cause of death in women [16]. Many factors have been implicated in breast cancer risk including, lifestyle, especially diet [17], estrogen levels [18], oxidative and carbonyl stresses [19]. Reactive oxygen species can attack every component of the cell, causing damage to the surrounding tissues with subsequent increased risk of various cancers [20]. A number of enzymes in the human body including, GLO1, act via free radical detoxification and protecting from genotoxic damage [21].

GLO1 gene polymorphism has been reported in some diseases other than breast cancer including, diabetes mellitus and its complications [22, 23], epilepsy [24] and autism spectrum disorder [25]. GLO1 CC genotype of (rs4746) polymorphism was associated with significantly higher prevalence of peripheral vascular and cardiovascular diseases in hemodialysis patients [26]. In human leukemia cells, GLO1 is involved in apoptosis resistance to antitumor agents and the use of GLO1 inhibitors prepare the cells to chemotherapeutic agents [27].

Although, GLO1 mRNA expression has been reported in several types of cancers, interestingly, no study has focused on the association between this gene polymorphism and its expression in breast cancer patients in Egypt. Moreover, their clinical role in breast cancer is poorly understood. Therefore, we analyzed for the first time GLO1 gene polymorphism and expression in Egyptian women with breast cancer.

In the present study, the frequency of AA genotype and A allele of GLO1 were significantly higher in the group of breast cancer patients compared to controls. In addition, upregulation of GLO1 mRNA was observed in breast cancer patients compared to controls. We established a strong correlation between up-regulation of GLO1 mRNA and A allele of GLO1 with higher stages of breast cancer. No correlation was observed between GLO1 gene polymorphism or expression with different breast cancer subtypes.

Our results go in line with Germanová et al. [16] who found that, the risk of breast cancer associated with the GLO1 C allele significantly increased with the increase in the tumor stage. Fonseca-Sánchez et al [28] reported higher GLO1 expression in breast cancerous tissue and this increase was associated with higher GLO1 protein levels and confirmed a strong correlation with tumor grade. They also found no correlation with hormonal receptor status or HER2-positive tumors.

Santarius et al [29], reported amplification of the GLO1 gene in 22% of breast tumors. By the contrary, Zhang et al demonstrated overexpression of GLO1 in HER2-positive breast tumors [30]. The change in GLO1 mRNA expression has been previously reported in several human cancers including cancer breast, cancer colon and in malignant melanoma [12,31,32].

In the above mentioned studies, no correlation was done between GLO1 gene polymorphism and expression in breast cancer patients, which we have proven for the first time in the present work.

With respect to GLO1 activity, we found that patients with poorly aggressive, less invasive tumors had a significantly lower enzymatic activity than highly aggressive, more invasive tumors. Non-statistically significant difference in GLO1 activity was observed among different subtypes. GLO1 A variant was associated with a high enzyme activity.

In agreement with us, higher GLO1 activity was observed in cancerous tissues, such as breast cancer [30], kidney tumors [33] and prostatic cancer [34].

By the contrary, Antognelli et al [35] demonstrated that, the presence of GLO1 polymorphism may be associated with decrease in GLO1 activity with subsequent increase in the breast cancer risk. Sakhi et al [36] analyzed GLO1 polymorphism in diabetic patients and reported no association between this SNP and enzyme activity. In human urogenital cancer [37], GLO1 activity was decreased with subsequent increase in AGEs. The difference between our results and others might be due to different ethinity.

The upregulation of GLO1 mRNA and the increase in its enzyme activity represents a defense response of tumor cells to the stress created by elevated cellular MG as a result of glycolytic adaptations to cancer (Warburg effect) [38].

GLO1 enzyme is involved in the detoxification of MG, one of the most potent oxidative stress precursor generating ROS and reactive nitrogen species [21]. If MG was accumulated, it may react directly with DNA, RNA, and proteins and stimulate cellular apoptosis [39]. Indirectly, it may cause glycation of nucleotides to form AGEs which produces cytotoxicity and DNA-protein cross-link [28]. Tesařová et al [19] reported higher serum levels of AGEs in breast cancer patients with stages III-IV than in patients with stages I-II.

AGEs are formed during the Maillard reaction. In Maillard reaction, non-enzymatic glycation of proteins, lipids or nucleic acids occur. This alteration in the protein results in change in the enzyme activity [40], decreases ligand binding, extracellular matrix proteins cross-linking causing stiffening and immunogenicity [41]. Moreover, protein glycation has been associated with several diseases as cardiovascular diseases [42], nephropathy [43], neuropathy and cancer [44].

AGE formation can result in malignant transformation due to increased free radical activity causing damage to the cell membranes and gene mutations [45]. AGEs exert their effects by binding to the receptor for advanced glycation endproducts (RAGE). RAGE is over expressed on vascular cells [46], activated immune cells [47] and cancer cells [48]. Moreover, its expression is induced under pathological conditions, by external stress, hypoxia, ROS [49], pro-inflammatory mediators, high glucose, and by AGE itself [50]. RAGE stimulation triggers the activation of the key mediators of the proliferation and inflammation, e.g. p21ras, MAP kinases, NF-kappaB and cdc42/rac. This results in increased cell proliferation and tumor growth through the production of ROS [51] and change in the transcription factors and in the gene expression [52]. Tumor metastasis occurs as a result of stimulation of cell migration and invasion.

Matrix metalloproteinases (MMPs) stimulated by AGEs plays an important role in cancer progression and invasion through degrading the connective tissue and the basement membrane [53]. AGE-RAGE binding stimulates release of growth factors and pro-inflammatory cytokines with changes in cell shape, cell survival, stress responses and apoptosis, through activation of phosphoinositol-3 kinase (PI3K) [52], protein kinase C (PKC), oncogenic Ras, and members of Rho/GTPase signaling pathways [54].

In conclusion, the findings of this study provide evidence that, GLO1 C332C genetic polymorphism results in upregulation of GLO1 mRNA and increase in the enzyme activity. Amplification of GLO1 may be involved in increasing the risk of breast cancer as well as its progression from localized to advanced. Furthermore, serum GLO1 enzyme activity may serve as noninvasive biomarkers for breast cancer diagnosis.

## SUBJECTS AND METHODS

### Participants

This study included 100 apparently healthy females as control group (mean age 53.9 ±7.3 years) and 200 females suffering from breast cancer diagnosed at the Department of Pathology, Zagazig University, Egypt by formalin-fixed paraffin-embedded tissue samples through biopsies and mastectomies. Breast cancer patients were then grouped into two groups: poorly aggressive, less invasive group which included 100 females (mean age 53.50±7.24 years) in the early stages of breast cancer (stage I & stage II) and highly aggressive, more invasive group which included 100 females (mean age 55.85±6.24 years) with advanced breast cancer (stage III & stage IV). Breast cancer patients were divided based on their TNM classification.

Breast cancer patients were classified according to their immunohistochemistry surrogates into the following subtypes: luminal A (ER+, PR+ and Her2−), luminal B (ER+, PR−/PR and Her2−), Triple Negative Breast Cancer (TNBC) (ER−, PR− and Her2−) and Her2+ (ER−, PR− and Her2+). We excluded all patients with any other diagnosed cancer, chronic renal failure, diabetes mellitus and any disease other than breast cancer from the study, as they may affect the study parameters. Patients taking antioxidant drugs treatment were also excluded. None of the patients received anti-neoplastic therapy before the study. Demographic and clinical characteristics of all participants were collected and presented in Table 1. A written informed consent was obtained from all participants.

### Samples collection

Blood samples were collected after overnight fasting. For genetic analysis, blood was collected in heparinized tubes and stored at -20°C till use. For biochemical assay, blood was collected in tubes without anticoagulant. The samples were centrifuged at 3500 rpm for 10 minutes. Serum was stored at -80°C for GLO1 enzyme activity assay.

### Genotyping for GLO1 C332C polymorphisms

Genomic DNA was isolated from the blood using the High Pure PCR Template Preparation Kit (Roche Diagnostics, GmbH, Mannheim, Germany) according to manufacturer's protocol. GLO1 C332C genotyping was performed using PCR-restriction fragment length polymorphism (PCR-RFLP) as previously described by Groener et al [13] with some modifications. Amplification was performed using a PTC-100 thermal cycler (MJ Research Inc, Watertown, Massachusetts). The primer sequences used for amplification were as follow: GLO1 forward, 5′-AGA CCA TGC TAC GAG TGA AG—3′ and reverse, 5′- TCC AGT AGC CAT CAG GAT CT-3′. The reaction mix of the total volume of 50 μl included 50 ng of genomic DNA, 20 pmoles of each primer, 100 mM each of dNTP, 1.5 mM of MgCl2, 10 mM Tris HCl pH 8.3, 50 mM of KCl and 1U of Taq DNA polymerase (SIGMA Chemical Co, St. Louis, Missouri, USA).

The following cycling conditions were used: 95°C for 5 min, and then 35 cycles of 95°C for 40 s, 57°C for 30 s and 72°C for 30 s, with a final extension at 72°C for 10 min. PCR products for GLO1 were digested overnight at 37°C, using Bsa I restriction enzyme (New England Biolabs Inc., Beverly, MA). The amplified products were separated by 2% agarose gel electrophoresis stained with ethidium bromide and visualized in UV light. Samples yielding 414 bp fragment indicate the presence of wild type (C332C) while 227 and 187 bp long fragments indicate the presence of mutant (A332A). The presence of 414, 227 and 187 bp long occurs in case of (C332A).).

### Glyoxalase 1 expression analysis

#### RNA isolation and cDNA Synthesis

Total RNA was isolated from the blood using Total RNA Purification Kit (Thermo Fisher Scientific) according to the manufacturer's instructions. RNA was reverse-transcribed using High-Capacity cDNA Reverse Transcription Kit (Thermo Fisher Scientific) according to the manufacturer's guidelines.

#### Real time PCR of GlO1

Real-time PCR was performed using the LightCycler^®^ 480 System (Roche Molecular Diagnostics, Germany). The primer sequences used were as follow (Metabion, Munich, Germany): for GLO1 F 5`-CCCCAGTACCAAGGATTTTCT-3`, R 5`-TGGGAAAATCACATTTTTGGA-3`; for β-actin (housekeeping gene) F: 5`- CCAACCGCGAGAAGATGA-3`, R: 5`CCAGAGGCGTACAGGGATAG 3`. PCR was performed in a total volume of 20 ul containing 5 ul cDNA template, 10 ul 2X SYBR^®^ Green PCR Master Mix (Applied Biosystems; Darmstadt, Germany) and 1 ul of each primer. The following cycling conditions were used: initial activation at 95° for 10 min, denaturation at 95°C for 10 s, 60°C for 27 s, 72°C for 3 s and lastly, at 40°C for 10 s. GLO1 expression was determined using the 2-ΔΔCt method [14].

### GLO1 enzyme activity assay

Serum GLO1 activity was measured with continuous spectrophotometric technique [15]. In brief, the reaction mixture of a total volume 2.4 ml contained 37.5 μmol phosphate buffer pH 7.4, 20 μmol Mg Cl_2_, 58 μmol methylglyoxal, 10 μmol reduced glutathione and 2 μmol dithiobisnitrobenzoic acid (SIGMA Chemical Co, St. Louis, Missouri, USA). Incubate for 2 min at 25^°^C then the rate of absorbance was recorded at 412 nm. Then add 0.1 ml of serum. The change in the absorbance was recorded and the results were expressed as μmol of GSH liberated per minute.

### Statistical analysis

Analysis of data was performed using SPSS. 17 software (SPSS, Inc., Chicago, IL, USA). Analysis of variance (ANOVA) test and student's t-test were used for comparison between groups. The chi square (x2) test was used to determine differences in the frequencies of genotypes and alleles between patients and controls. The odds ratio (OR) and 95% confidence interval (95% CI) were calculated to detect disease susceptibility. P value < 0.05 was considered significant.
